# Recurrent invasive lobular carcinoma presenting as a ruptured breast implant

**DOI:** 10.2478/v10019-011-0032-5

**Published:** 2011-10-08

**Authors:** Maikel Botros, Kenneth Chang, Robert Miller, Sunil Krishnan, Matthew Iott

**Affiliations:** 1 East TN State University, Quillen College of Medicine, Johnson City, Tennessee, USA; 2 Boston College, Chestnut Hill, Massachusetts, USA; 3 Department of Radiation Oncology, Mayo Clinic, Rochester, Minnesota, USA; 4 Department of Radiation Oncology, the University of Texas MD Anderson Cancer Center, Houston, Texas, USA

**Keywords:** breast cancer, invasive lobular carcinoma, breast implant, rupture

## Abstract

**Background:**

For years, the treatment for invasive lobular carcinoma (ILC) has been mastectomy secondary to the lack of studies investigating the efficacy of breast conservation therapy on patients afflicted with ILC and due to the lack of long-term follow up investigating locoregional recurrence in this patient population. In this article we report the clinical course of a patient diagnosed with ILC.

**Case report:**

We describe the case of a 50-year-old woman with stage IIB (T2N1M0) ER/PR positive right breast ILC who underwent a right modified radical mastectomy, postoperative chemotherapy, a prophylactic left simple mastectomy with bilateral breast reconstruction and tamoxifen. Approximately 12 years later, she presented with a deflated breast implant and recurrent breast cancer with metastatic spread. She received palliative radiotherapy then palliative chemotherapy. Unfortunately, she succumbed to the cancer less than a year after being diagnosed with metastatic disease.

**Conclusions:**

This may be the first case report of a ruptured breast implant presenting at the same time as the diagnosis of recurrent breast cancer.

## Introduction

There were an estimated 209,000 newly diagnosed cases of breast cancer in 2010. In the United States, breast cancer is the most common cancer among women and the second leading cause of cancer death for this cohort.^1^ Invasive ductal carcinoma (IDC) comprises 70–85% of diagnosed breast cancers.^2^ The second most common histological type of breast cancer is invasive lobular carcinoma (ILC), which makes up 8–14% of invasive cancer cases.^3^ While it is thought that the prognosis of ILC is generally similar to that of IDC, given similar histological grades, differences between the two types do exist.^3^,^4^ These differences may include presenting as an indistinct thickening as opposed to a discrete nodule, having less microcalcifications and decreased density of the mass seen on mammography, as well as, the likelihood of having multicentric and/or bilateral disease. Metastatic dissemination of disease also varies.^4^ In addition, the management of IDC and ILC has differed.

For years, the treatment for ILC has been mastectomy secondary to the lack of studies investigating the efficacy of breast conservation therapy on patients afflicted with ILC and due to the lack of long-term follow up investigating locoregional recurrence in this patient population. In this article, we report the clinical course of a patient diagnosed with ILC. She underwent bilateral mastectomy followed by breast reconstruction. Almost 12 years later, she suffered a breast implant rupture. Work up for the rupture resulted in her eventual diagnosis of recurrent breast cancer. To our knowledge, this is the first reported case of breast cancer recurrence presenting as a ruptured implant. It is possible that the rupture was secondary to cancer growth.

## Case report

A 50-year-old post-menopausal woman was diagnosed with a stage IIB (T2N1M0) right breast cancer and subsequently years later developed an unusual recurrence that was diagnosed following rupture of one of her breast prostheses. Following initial diagnosis, she underwent a right modified radical mastectomy and review of surgical pathology revealed a grade 4 (of 4) invasive lobular carcinoma, nuclear grade 2 (of 3), forming a 2.2 × 2 × 1.8 cm mass. No definite vascular invasion was noted apart from the central tumor mass, although lobular carcinoma in-situ with extension into adjacent ducts was seen. Lactiferous ducts beneath the nipple showed pagetoid spread of carcinoma cells. One of 14 right axillary lymph nodes was positive for metastatic involvement with focal extranodal extension of disease. Tumor cells were ER/PR positive. Following surgery, she received six months of chemotherapy with cyclophosphamide, methotrexate and 5-fluorouracil (CMF). A subsequent prophylactic left simple mastectomy with bilateral breast reconstruction was performed 4 months following completion of chemotherapy. Approximately 4 months after surgery, tamoxifen therapy was started and administered for 5 years. Of note, this patient underwent a total abdominal hysterectomy and bilateral salpingo-oophorectomy a year after breast reconstructive surgery.

The patient had regular follow up without evidence of disease recurrence. Approximately 12 years after her breast reconstructive surgery, she developed a deflated right breast implant. She was scheduled for bilateral implant exchange surgery. During preoperative evaluation, she was found to have evidence of mitral valve regurgitation due to a flail mitral valve posterior leaflet, and subsequently underwent mitral valve repair. The cardiothoracic surgeon informed the patient that her sternum was found to be “somewhat mushy” during her sternotomy.

**FIGURE 1:**
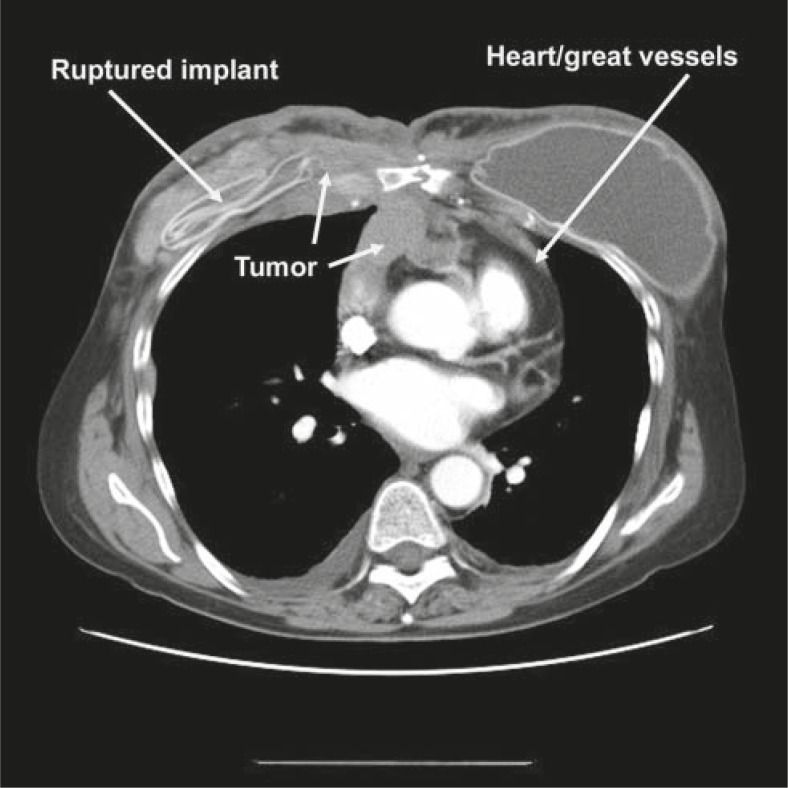
Deflated right breast implant with associated recurrent cancer infiltrating the chest wall and thorax.

**FIGURE 2:**
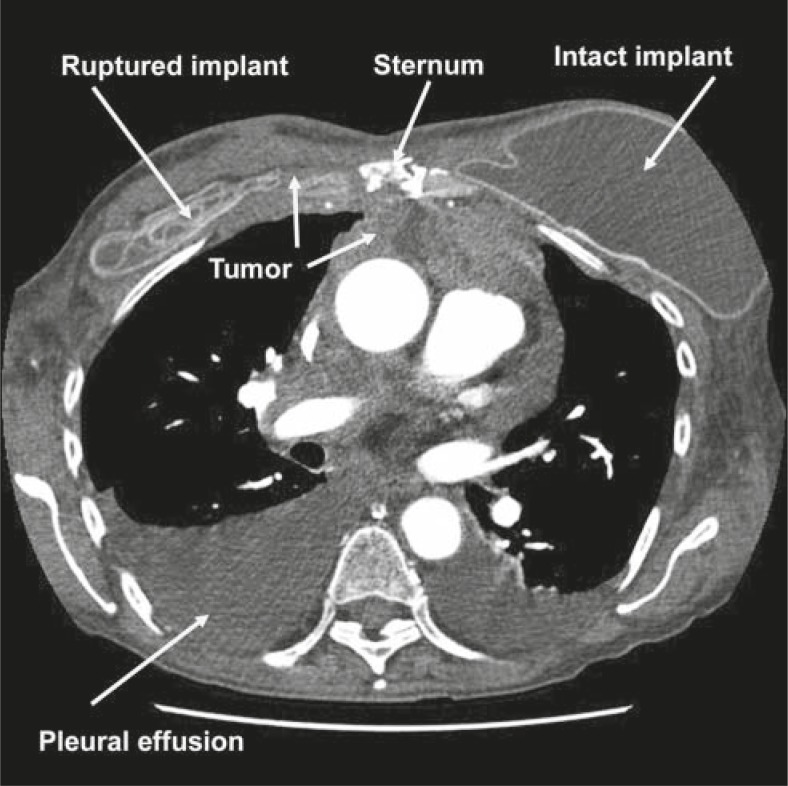
Disease infiltrating the mediastinum from recurrent breast cancer.

About 5 months after open heart surgery, the patient had developed a neck lump and back pain. Imaging studies with CT revealed postoperative mastectomies with implants. However, the right breast implant was ruptured with extensive soft tissue mass and nodularity involving the anterior chest wall, predominantly anterior to both sides of the sternum but slightly more marked on the right with subcutaneous nodularity throughout the right mastectomy site (Figure 1). This was noted to be inseparable from the adjacent pectoralis muscles along with right subpectoral adenopathy and right neck base adenopathy consistent with tumor recurrence. The anterior chest wall mass extended posteriorly through the chest wall into the hemithorax and was also associated with internal mammary adenopathy. Partial lytic lesions were seen in the mid sternum. In addition, there was bulky anterior mediastinal adenopathy and tumor extending inferiorly along the anterior pericardium and anterior to the right atrium and right ventricle as well as to the root of the aorta (Figure 2). Nodularity was noted in the right upper lung pleura and left lung base pleura. There were bulky soft tissue masses in both costophrenic angles. Tumor nodularity was noted anterior to the liver representing peritoneal implants. Skin thickening was noted over both anterior chest walls but greater on the right. Bony metastases were noted in the T5 and L1 vertebral bodies, the right temporal bone of the skull, and the right anterior iliac bone.

The patient underwent a T10 vertebroplasty and then subsequent palliative radiotherapy to T8 through L1 vertebral bodies. During palliative radiotherapy, she developed right hip pain and was found to have a destructive metastasis in the right femoral head and neck requiring surgery with a right hip replacement followed by palliative radiotherapy to bilateral hips and the right femur. She went on to receive palliative chemotherapy but ultimately expired from disease progression approximately 11 months following diagnosis of metastatic disease.

## Discussion

Breast cancer recurrence is a challenging event that is associated with morbidity and shortened survival.^5^,^6^ Herein, we described the course of a patient who developed locoregional and distant metastases of ILC. Although ILC makes up a minority of breast cancer cases, its incidence is believed to be on the rise.^7^ These tumors are less likely to result in a reactive process and are also less likely to have microcalcifications.^8^,^9^ Thus, they are occasionally missed on screening mammography and clinical examination.

As reported in previously published studies, the prognosis of ILC as compared to IDC has ranged from either superior, the same, or poorer.^8^ Although, most recent studies confirm that the prognosis of ILC is indeed similar to that of IDC.^9^–^12^ Sastre-Garau *et al.* examined the difference in overall survival, incidence of local or distant recurrence, disease free interval, rate of metastasis and pattern of metastatic distribution among patients who were diagnosed with ILC, ILC-IDC mixed histology, and non-ILC. They found that univariate and multivariate analysis did not reveal any statistically significant difference between these groups regarding overall survival, recurrence, disease free interval, or the frequency of metastasis. However, the pattern of distribution of metastases did differ among the groups. ILC patients were more likely to have disease disseminate to bone rather than the lungs or pleura as seen more often with IDC. ILC patients were also more likely to have distant disease in the peritoneum, gynecological tract, and gastrointestinal tract.^10^ This pattern of metastatic dissemination has also been reported in several previously published studies.^10^,^13^–^15^ Our patient did indeed become afflicted with this pattern of metastatic distribution as she had multiple bony metastases as well as peritoneal metastasis.

According to the current consensus, treatment for ILC may include breast conservation surgery when surgical margins are adequate along with subsequent radiation therapy.^7^ A study published by Santiago *et al.* compared long-term outcomes for women who underwent breast conservation surgery for either early stage ILC or IDC and found the results be similar between the groups.^16^ Although due to difficulty in localization and margin detection associated with this particular tumor, the rates of mastectomy in patients afflicted with ILC have been higher than that for IDC. However, treatment trends show that there is movement from aggressively treating ILC with mastectomy to breast conservation.^17^ If a tumor can be removed by lumpectomy with negative margins, then lumpectomy and radiation therapy is warranted.^7^

It is important to point out that our patient did not undergo post-mastectomy irradiation during treatment of her primary tumor. Studies that date as far back as the early 1970s have shown that the use of post-mastectomy adjuvant radiation decreases the incidence of locoregional recurrence by two thirds.^18^ Two published studies have also shown that the use of adjuvant irradiation has a positive effect on survival rates, with the study by Overgaard, *et al.* showing a statistical significance in overall survival in high risk post-menopausal woman diagnosed with brest cancer.^19^, ^20^

As for the risk of recurrence, Recht *et al.* examined factors associated with locoregional failure (LRF) in breast cancer patients from four randomized ECOG trials. The patients in this study had undergone mastectomy, chemotherapy with or without tamoxifen, and without radiation. These patients fit the treatment scheme as that of the patient presented herein. At 10 years, the risk of LRF ± distant failure (DF) was reported as 12.9% for patients who had one to three positive nodes at the time of their primary cancer (like the patient presented). It was estimated that 80 % of the patients who had an isolated recurrence and LRF ± DF were diagnosed within 5 years of their primary cancer. The study does not mention the histological types of tumors in this patient population.^21^ Moreover, the presentation of the tumor recurrence is accountable for the clinical outcome of the patient. Willner *et al.* found that survival rate decreased significantly if recurrence involved multiple nodules, with a 5-year survival rate of 12%.^22^

Treatment of locoregional recurrence involves an attempt to clear all local disease. Studies reveal that local control is the most important treatment factor. Interestingly, the optimal local treatment of recurrences requires not only excision but also the use of adjuvant radiotherapy. The use of excision alone has reported failure rates of 57%–76%.^22^

There are several known and reported complications of breast implants including contracture of tissue surrounding the breast implant and implant rupture. Implant rupture is thought to be associated with the age of the implant.^23^,^24^ There has also been much speculation about the risks of breast cancer development and the prognosis of diagnosed breast cancer in patients with breast implants. Reasonably, it has been thought that the uncontrolled and ubiquitous phenomenon of gel bleed, which is the diffusion of silicone across the implant’s intact envelope, can result in chronic inflammation. The chronic inflammation would thus increase the potential for cancer development.^25^ This theory has not been proven clinically in patients. More importantly, studies did find that breast implants impede visualization of breast tissue during screening mammography.^25^,^26^ As a result, it is feared that implants may delay diagnosing of breast cancer, leaving the patients with an advanced stage tumor. This delay has been reported by several studies, which confirmed that patients with implants were diagnosed with more advanced breast cancer.^26^,^27^ These studies had small patient populations. Two large cohort studies have found that there is no clinical association that would suggest that patients with implants have worse prognosis upon diagnosis of their cancer.^25^,^28^–^30^ The Los Angeles study showed that there was no difference in stage of breast cancer between women with breast implants and women without implants.^29^ The Alberta study showed that there was no significant difference in survival rates between cancer patients who had implants and those without implants.^30^

Lastly, it is important to point out that these studies have focused on the development of primary breast cancer after cosmetic breast augmentation. There is little known about the development of breast cancer in patients who had undergone reconstructive surgery.^25^ Petit *et al.* compared 146 breast cancer patients who had undergone reconstructive surgery to 146 matched control patients who underwent mastectomy but not reconstructive surgery. The study showed that patients who had implants were at a lower risk of death due to breast cancer, distant metastasis, and local recurrence. These patients did not have an increased risk of developing a second primary breast cancer.^31^ In addition, there is insufficient evidence that silicone breast implants may cause other cancers.

Interestingly, there has been one case report in which a primary desmoid tumor was associated with a silicone implant rupture.^32^ To our knowledge this is the first reported case in which a ruptured breast implant appeared at the same time as cancer recurrence. Perhaps the uncontrolled growth of the recurrent cancer may have contributed to the implant rupture.
